# Visualization and Analysis of Neuromuscular Junctions Using Immunofluorescence

**DOI:** 10.21769/BioProtoc.5076

**Published:** 2024-10-05

**Authors:** You-Tian Hsieh, Show-Li Chen

**Affiliations:** Graduate Institute of Microbiology, College of Medicine, National Taiwan University, Taipei, Taiwan

**Keywords:** Neuromuscular junction, Acetylcholine receptor, Immunofluorescence assay, α-bungarotoxin

## Abstract

The neuromuscular junction (NMJ) is an interface between motor neurons and skeletal muscle fibers, facilitating the transmission of signals that initiate muscle contraction. Its pivotal role lies in ensuring efficient communication between the nervous system and muscles, allowing for precise and coordinated movements essential for everyday activities and overall motor function. To provide insights into neuromuscular disease and development, understanding the physiology of NMJ is essential. We target acetylcholine receptors (AChR) by immunofluorescence assay (IFA) with α-bungarotoxin (BTX; snake venom neurotoxins binding to AChR) to visualize and quantify the NMJ. Changes in AChR distribution or structure can indicate alterations in receptor density, which may be associated with neuromuscular disorders or conditions that affect synaptic transmission. This protocol provides the methodology for isolating and longitudinally sectioning gastrocnemius muscle for AChR-targeted IFA for confocal microscopy and performing quantitative analysis of NMJs.

Key features

• Visualizes and quantifies NMJs using α-bungarotoxin.

• Utilizes high-resolution confocal microscopy for detailed imaging.

## Graphical overview


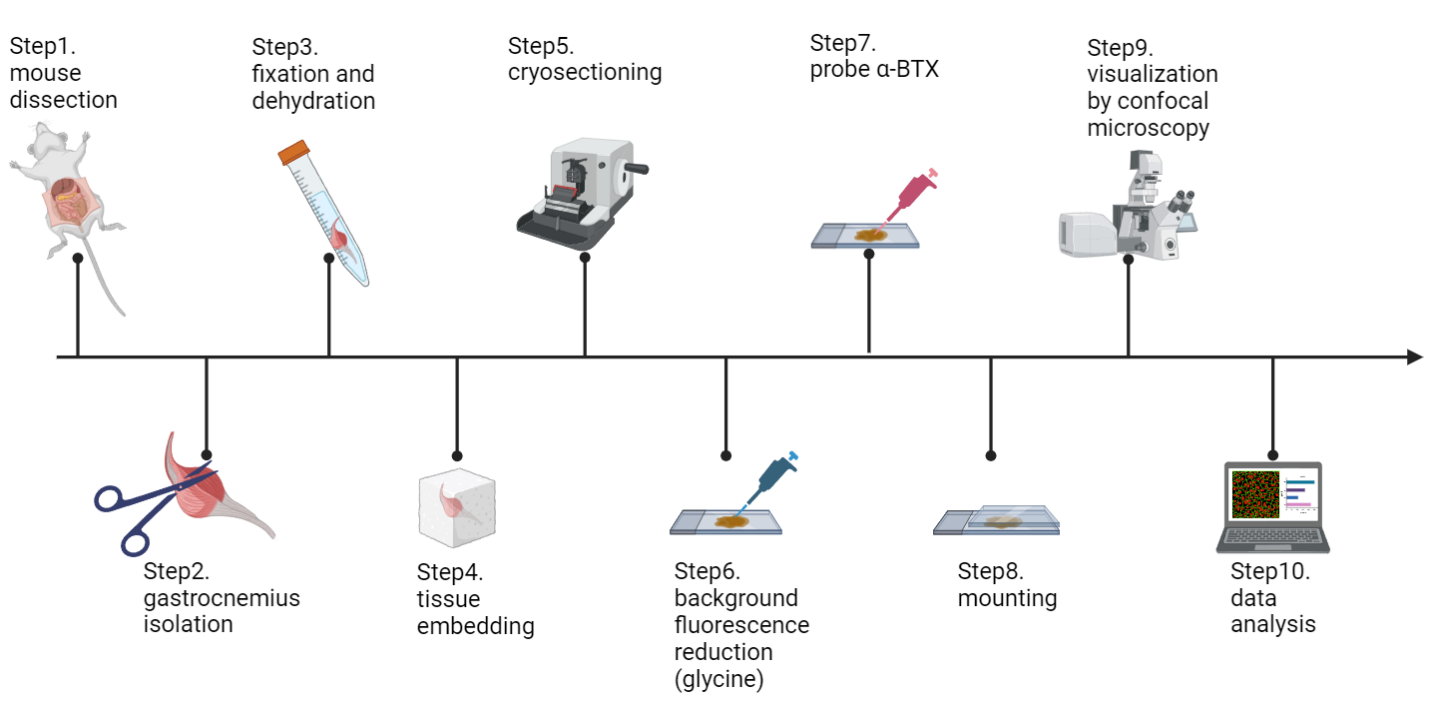


**Schematic workflow of this protocol (figure created in** **BioRender.com** )

## Background

The neuromuscular junction (NMJ) serves as a chemical synapse between the axon terminal of a motor neuron and the postsynaptic region on a muscle fiber [1]. At the NMJ, the motor neuron's axon terminal releases acetylcholine (ACh) into the synaptic cleft. This neurotransmitter then binds to nicotinic acetylcholine receptors (AChR) on the muscle fiber membrane, causing an influx of sodium ions and subsequent depolarization of the muscle cell membrane. This depolarization initiates an action potential that travels along the muscle fiber, ultimately leading to muscle contraction. The NMJ is vital for voluntary muscle movement and is precisely regulated to ensure accurate motor control [2].

Structural instability of NMJ can cause several neuromuscular diseases and may occur in the aged population to cause loss of muscle strength and mass [3]. For instance, we have recently found that the nuclear receptor interaction protein (NRIP) is an AChR-interacting protein, functioning as a scaffold to stabilize the AChR complex and playing a physiological role in the neuromuscular system [1]. Without NRIP, the efficacy of synaptic transmission and AChR clustering may decline, leading to the loss of nerve supply to NMJs (denervation) and impaired motor function, as observed in NRIP knockout mice (Figure 3C and D in Chen et al. [4]). Additionally, Lrp4 regulates the AChR clustering to guarantee proper NMJ assembly [5]. Conditional knockout of muscle Lrp4 causes a significant decline in AChR cluster size and number [6]. Besides muscle disorder, nerve injury also causes a decrease in AChR cluster and density. Researchers have used a sciatic nerve crush injury model in mice to simulate nerve damage and observed significant fragmentation of the NMJ and a notable decrease in the AChR cluster area [7].

NMJ analysis is a crucial tool in neuromuscular disease research. By combining high-resolution imaging with molecular studies, researchers gain a comprehensive understanding of NMJ physiology, which aids in developing effective treatments for amyotrophic lateral sclerosis (ALS), spinal muscular atrophy (SMA), and other neuromuscular diseases. Key visualization techniques include immunofluorescence assays (IFA) with confocal microscopy, electron microscopy, and super-resolution microscopy. IFA with confocal microscopy is preferred for its ability to produce high-resolution 3D images, enabling detailed examination of NMJ structures. α-bungarotoxin, a snake venom toxin, is commonly used to bind acetylcholine receptors for precise visualization. Despite its origin, α-bungarotoxin is safe in labs due to non-toxic concentrations. Confocal microscopy enhances IFA by reducing background noise and increasing image clarity, surpassing traditional fluorescence microscopy. While electron microscopy offers ultra-high resolution, its extensive sample preparation makes it less practical. Super-resolution microscopy provides nanometer-scale visualization but is limited by the complexity and specialized equipment needed. Overall, IFA with confocal microscopy balances high-resolution imaging with ease of use and specificity, making it indispensable in neurobiological research and treatment development for neuromuscular diseases.

## Materials and reagents

1. High-profile microtome blades (Leica, catalog number: 14035838383)
2. Glass insert 70 mm (Leica, catalog number: 14047742497)
3. Micro slides (Muto Pure Chemicals, catalog number: GA13-7626R)
4. O.C.T (VWR, catalog number: 95057838)
5. Aluminum foil (Kirkland, catalog number: RK611)
6. 10× phosphate-buffered saline (PBS) (Omics Bio, catalog number: IB3012)
7. Sucrose (Sigma-Aldrich, catalog number: S0389-500G)
8. Glycine, powder (Omics Bio, catalog number: BT5031)
9. Paraformaldehyde powder (Sigma-Aldrich, catalog number: P6148-500G)
10. Alexa-594-conjugated α-bungarotoxin (Life Technologies, catalog number: B13423)
11. DAPI fluoromount-G (SouthernBiotech, catalog number: 0100-20)
12. Microscope cover glass (Shinetech Inc, catalog number: GA11-2450)
13. 1.5 mL clear microcentrifuge tubes (Corning, catalog number: MCT-150-C)
14. 2,2,2-tribromoethanol (avertin) (Sigma-Aldrich, catalog number: T48402-5G)
15. Horse serum (Gibco, catalog number: 16050-114)
16. BSA albumin fraction V (BioFroxx, catalog number: 4240GR100)
17. Anti-synaptophysin primary antibody (Abcam, catalog number: ab32127)
18. Anti-neurofilament primary antibody (Abcam, catalog number: ab8138)
19. 488-conjugated donkey anti-rabbit secondary antibody (Jackson ImmunoResearch, catalog number: AB_2340620)

**Solutions**
1. 1× PBS (see Recipes)
2. 4% PFA (see Recipes)
3. 2.5% avertin (see Recipes)
4. 0.1 M glycine solution (see Recipes)
5. 30% sucrose (see Recipes)
6. Blocking buffer (see Recipes)

**Recipes**
1. **1× PBS**
  **Table:** 

Reagent	Final concentration	Amount
10× PBS	n/a	200 mL
Deionized H_2_O	n/a	1800 mL
Total	n/a	2000 mL
*Note: The 1× PBS solution should be prepared fresh and used within seven days.*
2. **4% PFA**
  **Table:** 

Reagent	Final concentration	Amount
Paraformaldehyde powder	4%	4 g
1× PBS	n/a	100 mL
Total	n/a	100 mL
*Note: To prepare 4% PFA solution, add 4 g of paraformaldehyde powder to 90 mL of 1× PBS, stir, and heat to 60–65 °C until dissolved. Add a few drops of 5 N NaOH to clear the solution (5 N NaOH is used to facilitate the dissolution of paraformaldehyde powder), cool to room temperature, add 1× PBS to 100 mL, and adjust the pH to 7.4. The solution can be prepared in advance and stored in a -20 °C freezer for long-term storage. Thaw the solution 30 min before use to ensure it is fully liquefied.*
3. **2.5% avertin**
  **Table:** 

Reagent	Final concentration	Amount
2,2,2-tribromoethanol	2.5%	250 μL
1× PBS	n/a	9.75 mL
Total	n/a	10 mL
*Note: The 2.5% avertin solution can be prepared in advance and stored in a 4 °C refrigerator for up to one month.*
4. **0.1 M Glycine solution**
  **Table:** 

Reagent	Final concentration	Amount
Glycine powder	0.1 M	0.75 g
1× PBS	n/a	10 mL
Total	n/a	10 mL
*Note: To prepare a 0.1 M glycine solution, add 0.75 g of glycine to 10 mL of 1× PBS in a 15 mL conical centrifuge tube and shake until the solution is clear. There is no need to adjust the pH of the solution. The solution can be prepared in advance and stored in a 4 °C refrigerator for up to two months.*
5. **30% sucrose**
  **Table:** 

Reagent	Final concentration	Amount
Sucrose	30%	15 g
1× PBS	n/a	50 mL
Total	n/a	50 mL
*Note: To prepare a 30% sucrose solution, add 15 g of sucrose to 50 mL of 1× PBS and shake until the solution is clear. The solution can be prepared in advance and stored in a 4 °C refrigerator for up to one month.*
6. **Blocking buffer**
  **Table:** 

Reagent	Final concentration	Amount
BSA albumin fraction V	2%	0.2 g
1× PBS	n/a	9.5 mL
Horse serum	5%	500 μL
Total	n/a	10 mL
*Note: To prepare the blocking buffer, add 500 μL of horse serum to 9.5 mL of 1× PBS in a 15 mL conical centrifuge tube, shake, and then add 0.2 g of BSA albumin fraction V into the solution. Shake until the solution is clear.*

## Equipment

1. P2, P200 and P1000 micropipettes
2. Laser scanning confocal microscope (Zeiss, model: LSM880)
3. Staining jar (Shineteh Instruments, catalog number: GB11-15)
4. Slide racks (Shineteh Instruments, catalog number: GB11-15S)
5. Liquid nitrogen insulation barrels (Shineteh Instruments, catalog number: IE1-D6000)
6. Cryostat (Leica, catalog number: CM3050)
7. 4 °C, -20 °C freezers [Sampo, catalog number: SR-C61G(K3)]
-80 °C freezers (Panasonic, catalog number: MDF-U74V)
9. Laminar flow cabinet (CHUAN, catalog number: TBH-520M)
10. Orbital shaker (TKS, catalog number: OS-701)
11. Disposable syringe with needle (Terumo, catalog number: MDSS01S2613)
12. Disposable syringe (Terumo, catalog number: MDSS20ES)
13. Iris scissors (Shineteh Instruments, catalog number: ST-S009)
14. Fine curve point tweezers (Shineteh Instruments, catalog number: ST-NO7)
15. Super fine point tweezers (Shineteh Instruments, catalog number: ST-NO3)
16. Iris forceps (Shineteh Instruments, catalog number: ST-I210)
17. Lab mat (Dogger, catalog number: D4EG-031055)
18. Falcon^TM^ 15 mL conical centrifuge tubes (Thermo Fisher, catalog number: 14-959-53A)
19. Immuno-pathology staining kit (PEOPLE LIFE TEK CO., LTD., catalog number: H198099910)

## Software and datasets

1. ImageJ image processing and analysis software (https://imagej.net/ij/)
2. ZEN microscopy software (https://www.zeiss.com/microscopy/en/products/software/zeiss-zen.html)
3. GraphPad Prism 8.0.2

## Procedure

- **Mouse dissection**
  1. Set up the dissection area with a 40 cm × 50 cm lab mat in a clean and well-lit workspace.
  2. Restrain the mouse as shown in Graphical overview. Insert the disposable syringe with a needle into the lower-right quadrant of the abdomen to avoid puncturing internal organs. Gently and slowly inject 0.5 mL of 2.5% avertin (Figure 1) (volume of avertin per mass: 0.2 mL/10 g body weight).
*Note: Alternatively, mice can be anesthetized with isoflurane using a gas anesthesia instrument, instead of using avertin.*
  3. Wait 5 min until the mouse is completely unconscious.
*Note: Tap the tail of the mouse to confirm that it does not respond to stimuli at all.*
  4. Gently place the mouse with its ventral side facing upward. It is recommended to pin the mouse to prevent movement during dissection. Use scissors to make a midline incision through the skin from the lower abdomen to the chest. Continue cutting through the muscle layers to expose the ribcage. Then, carefully cut through the ribs on both sides to open the chest cavity and expose the heart.
  5. Gently lift the heart using iris forceps and create a small incision (0.5–1.5 mm wide) in the right atrium with iris scissors. Insert a perfusion needle (connected to the perfusion syringe) into the left ventricle, ensuring the needle tip penetrates the ventricular wall to prevent leakage (Figure 2).
    **Figure 1.**
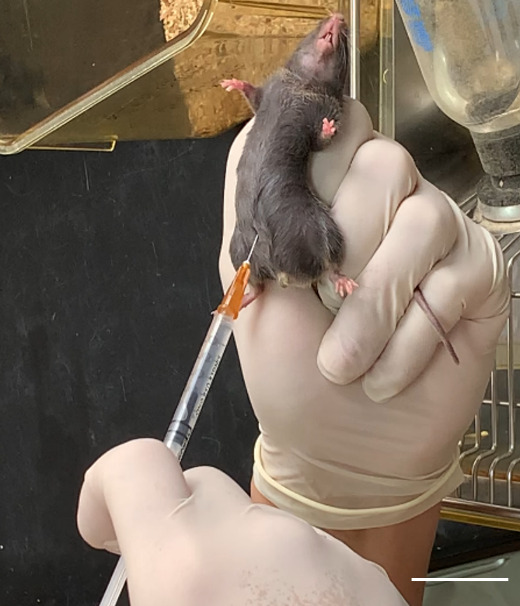
Avertin intraperitoneal injection. Gently restrain the mouse, insert the needle into the lower abdomen slightly to the right of the midline, and slowly inject 0.5 mL of 2.5% avertin solution. Scale bar, 3 cm.
    **Figure 2.**
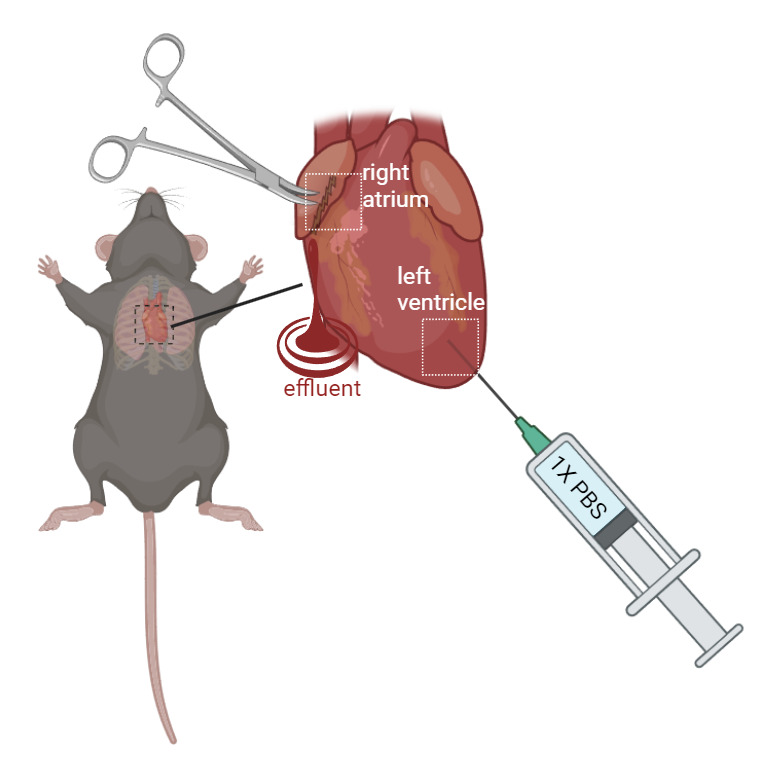
Schematic figure of mouse perfusion. Gently restrain the mouse. Create a 0.5–1.5 mm incision with iris scissors. Perfuse with 1× PBS at a steady rate (10–15 mL/min) using a perfusion syringe with a needle inserted into the left ventricle. The effluent will exit the heart from the incision at the right atrium. (created in BioRender.com)
  6. Begin perfusing with PBS at a steady rate (10–15 mL/min). This step clears the blood from the circulatory system. You will see the liver and other organs blanch as the blood is replaced with PBS.
  7. Continue perfusing until the effluent (fluid exiting the incision in the right atrium) runs clear, indicating that the blood has been adequately removed.
  8. Remove the skin and main organs in the abdominal cavity including stomach, liver, kidneys, small and large intestine, and spleen to facilitate the subsequent dissection steps.
  9. To expose the muscle and knee, remove the skin from the area around the hindlimb by lifting the skin with fine-point tweezers and cutting the fascia between the skin and muscle with iris scissors.
  10. Separate the hindlimbs from the body by cutting the knees with iris scissors.
  11. Incise the Achilles tendon at the ankle (Figures 3 and 4); then, use super fine point tweezers to pull the severed tendon toward the knee (Figures 3 and 5).
*Note: The Achilles tendon is the tendon shared by the soleus and gastrocnemius muscle.*
    **Figure 3.**
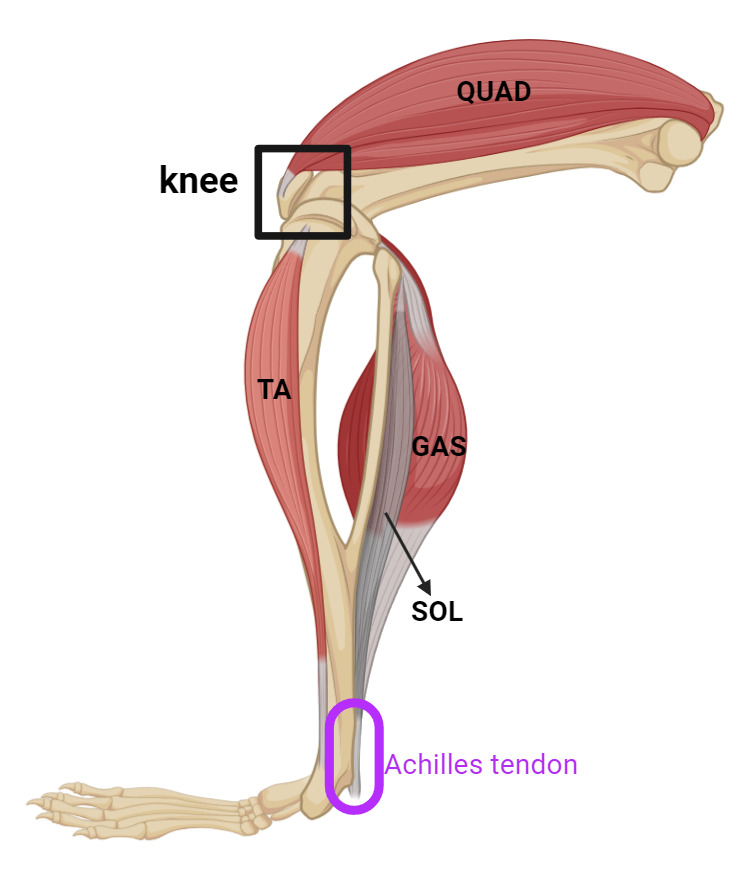
Schematic image illustrating the posterior view of the hindlimb of mice, highlighting key muscles and Achilles tendon. The knee is indicated with a black box, and the Achilles tendon is highlighted in purple at the back of the ankle. The quadriceps (QUAD) are located at the front of the thigh. The tibialis anterior (TA) runs along the front of the lower leg. The gastrocnemius (GAS) and soleus (SOL) muscles form the bulk of the calf. (created in BioRender.com)
    **Figure 4.**
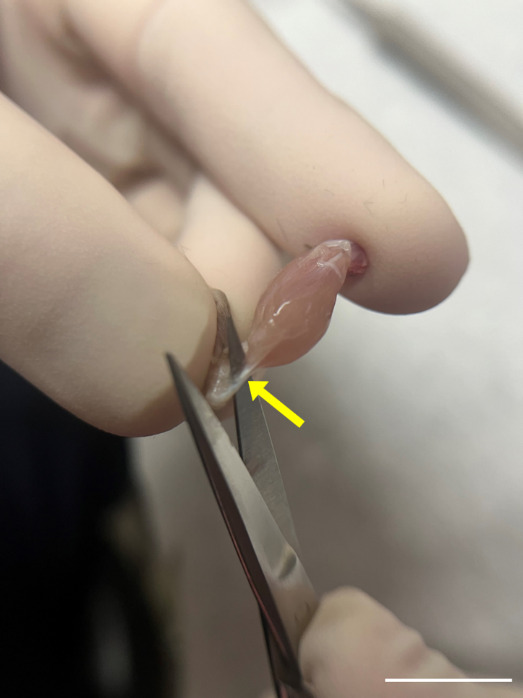
Incision of the Achilles tendon. Use iris scissors, carefully sever the Achilles tendon (yellow arrow). Scale bar, 1 cm.
    **Figure 5.**
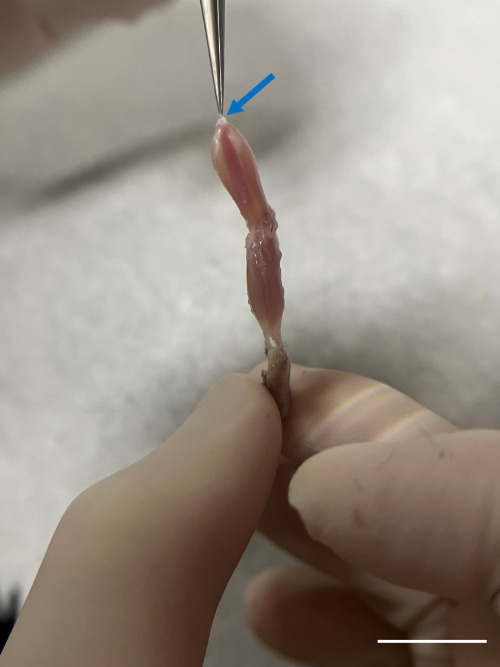
Precise tendon grasp for gastrocnemius isolation. Delicately grasp the severed tendon (blue arrow) using super fine point tweezers and gently draw it toward the knee. Do not pull too hard or too fast to prevent damaging the muscle structure. Scale bar, 1 cm.
  12. Cut the tendon in the knee region with iris scissors to separate the muscle from the hindlimb.
  13. Remove the soleus muscle and use fine curve point tweezers to lift the fascia, a thin transparent membrane that sits on the surface of the muscle, from the gastrocnemius muscle. Then, cut it with iris scissors (Figure 6).
*Note: The gastrocnemius muscle was chosen for the experiment because it is large enough for cryosectioning. Alternatively, soleus and tibialis anterior muscle are also acceptable (see General note section).*
    **Figure 6.**
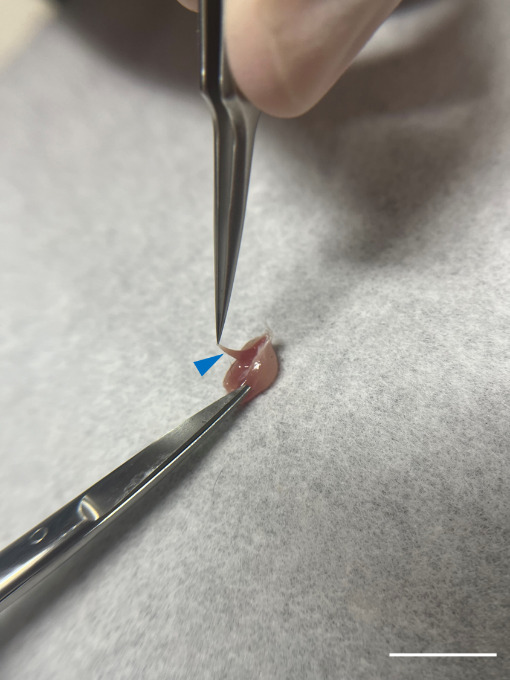
Detachment of soleus muscle. Carefully extract the soleus muscle (blue arrowhead) and remove any residual fascia surrounding the gastrocnemius muscle with iris scissors. Scale bar, 1 cm.
- **Preparation for cryosection**
  1. Subsequent to their separation from the mouse, submerge the gastrocnemius muscles in 4% paraformaldehyde in a 15 mL conical centrifuge tube and shake on the orbital shaker for 2 h at room temperature for fixation.
  2. Wash the muscle tissues three times with approximately 10 mL of 1× PBS in the 15 mL conical centrifuge tube, each time for a duration of 10 min, at room temperature.
  3. Incubate the muscle tissue with rotation in 10 mL of 30% sucrose in a 15 mL conical centrifuge tube at 4 °C overnight to facilitate dehydration.
  4. Place the muscle on a clean lab mat for subsequent experiments.
  5. Make a cube mold with aluminum foil (Figure 7) and fill it with O.C.T. compound until it is 70% full.
    **Figure 7.**
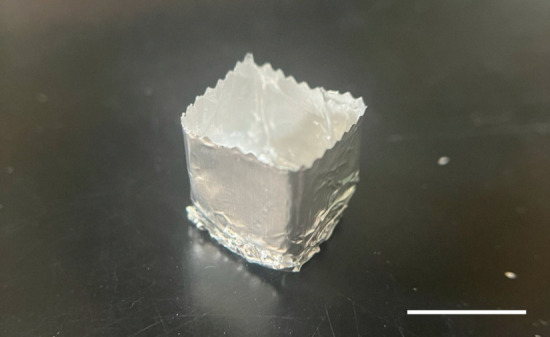
Representative image of a cube mold made from aluminum foil for embedding the muscle tissue in the O.C.T compound. Scale bar, 1 cm.
  6. Using forceps, carefully position the muscle tissue longitudinally in the center of the cube mold. Ensure that the entire tissue is submerged in the O.C.T compound.
*Note: It is strongly recommended to mark the side that aligns with the intended cutting plane to avoid sectioning in the wrong direction. The intended cutting plane should be parallel to the longitudinal axis of the muscle.*
  7. Using forceps, carefully hold the cube mold and immerse it into the liquid nitrogen in the liquid nitrogen barrel. To ensure there is no direct contact between the O.C.T. compound and the liquid nitrogen, do not submerge the mold with liquid nitrogen.
*Note: Placing the mold with dry ice, instead of liquid nitrogen, is acceptable for the O.C.T to solidify.*
  8. Once the O.C.T. compound has solidified, transfer it to a cryostat for immediate sectioning or store it at -80 °C for future use.
  9. Set the cryostat temperature to -20 °C and install a new or sharp cryostat blade and anti-roll plate.
  10. Remove the aluminum foil from the O.C.T.-embedded muscle tissue block and place it into the cryostat chamber.
  11. Mount the O.C.T. block on the specimen holder of the cryostat with the muscle tissue oriented longitudinally (parallel to the direction of sectioning).
  12. Trim the block face with a thickness of 50 μm using the cryostat blade until the muscle tissue is exposed and a smooth surface is achieved.
  13. Start sectioning the muscle tissue slowly and carefully into slices with a thickness of 30 μm each. Maintain a steady hand and consistent speed to produce even sections.
*Note: The reason for cutting such thick sections is to preserve the integrity of the muscle fibers and neuromuscular junctions (NMJs), which can be compromised in thinner sections. This preservation ensures that the fine details of the NMJ are more easily observable.*
  14. After ensuring the section is not curled or folded, attach the section with the pre-labeled slide. The section will adhere to the slide due to the temperature difference. (The slides are kept at room temperature throughout the entire procedure.)
  15. Continue cutting and collecting sections until you have the desired number of sections.
*Note: At least three sections should be placed on one slide.*
  16. After sectioning, wrap the tissue in aluminum foil and store it at -80 °C for future use.
- **Quenching (background fluorescence reduction)**
  1. Wash the slide carrying the sectioned tissue with approximately 250 mL of 1× PBS three times for 10 minutes each at room temperature to eliminate the O.C.T. compound. The slide-washing procedure entails placing the slides onto the slide rack, fully submerging the slide rack into 1× PBS in the staining jar, and then positioning the staining jar on the orbital shaker, shaking at 60 rpm. It is strongly recommended not to shake at speeds exceeding 60 rpm, as this may cause the tissue to detach from the slides.
  2. Take the slide out of the slide rack.
*Note: See the troubleshooting section about signal weakness when staining with antibodies for neurofilament or synaptophysin to visualize axonal innervation to NMJs.*
  3. Apply 200 μL of 0.1 M glycine solution onto each section and allow it to incubate for 40 min at room temperature, ensuring complete coverage of the tissue by the liquid. This step helps reduce tissue autofluorescence and quench free aldehydes left over from fixation with paraformaldehyde. This step does not require shaking.
  4. Dump the glycine solution from the slide.
  5. Place the slide back in the slide rack and submerge the slide rack in approximately 250 mL of 1× PBS in the staining jar.
  6. Place the staining jar onto the orbital shaker and shake at 60 rpm, three times for 10 minutes each at room temperature to eliminate any residual glycine.
- **Probe primary antibody (optional)**
  1. Take out the slide from the slide rack and place it in the immuno-pathology staining kit.
  2. Apply 200 μL of blocking buffer onto each section and allow it to incubate for 1 h at room temperature, ensuring complete coverage of the tissue by the liquid. This step reduces nonspecific binding.
  3. Dump the blocking buffer from the slide and place the slide in the immuno-pathology staining kit.
  4. Combine anti-synaptophysin primary antibody and anti-neurofilament primary antibody, each diluted at a 1/200 ratio in blocking buffer. Apply 200 μL of this solution onto each section.
  5. Incubate in an immuno-pathology staining kit overnight at 4 °C. This step does not require shaking.
*Note: The immuno-pathology staining kit must be humidified. Placing a wet paper towel in the immuno-pathology staining kit is feasible.*
  6. Dump the primary antibody solution from the slide.
  7. Place the slide back in the slide rack and submerge the slide rack in approximately 250 mL of 1× PBS in the staining jar.
  8. Place the staining jar onto the orbital shaker and shake at 60 rpm, three times for 10 min each at room temperature.
- **Probe with α-bungarotoxin and secondary antibody**
  1. Take out the slide from the slide rack and place it in the immuno-pathology staining kit.
  2. Apply 200 μL of Alexa-594-conjugated α-bungarotoxin diluted in 1× PBS at a 1/1,000 dilution onto each section and incubate for 2 h at room temperature in darkness. This step does not require shaking.
*Note: Add 488-conjugated donkey anti-rabbit secondary antibody to the α-bungarotoxin solution at a 1/1,000 dilution rate. There is no need to apply the secondary antibody if section D has not been conducted.*
  3. Remove the α-bungarotoxin solution and proceed to wash the slide three times at room temperature with approximately 250 mL of 1× PBS with shaking on the orbital shaker at 60 rpm for 10 min each time.
- **Mounting**
  1. Take out the slide from the slide rack and place it in the immuno-pathology staining kit.
  2. Add 20 μL of DAPI fluoromount mounting solution onto each section for nucleus staining.
  3. Hold the slide gently, then slowly lower the coverslip onto the slide by gradually tilting it down over the drop of mounting medium. This technique allows the mounting medium to spread evenly and avoids introducing bubbles once the coverslip is in place.
  4. Seal the edges of the coverslip with nail polish.
  5. Air-dry the slide by placing it in a laminar flow cabinet for 20 min.
- **Visualizing NMJs**
  1. Switch on the confocal microscope and ensure that the connected computer is also powered on.
  2. Open the Zen software on the computer. Once the software is running, activate the laser by following the manufacturer's guidelines.
  3. Mount the slide steadily on the stage of the microscope.
  4. Switch to *locate* mode.
  5. Use the 20× objective lens to scan the sample and identify the region containing the NMJ. The appearance of the NMJ is characterized by red, pretzel-like clusters, which extend less than 15 μm along the z-dimension.
  6. Switch to *Acquisition* mode within the Zen software.
  7. Configure the imaging parameters as detailed in Table 1. This includes settings for laser intensity, detector gain, and scan speed, among others.
    **Table 1.** Confocal microscopy image snapping condition

	αBTX	DAPI
Detection wavelength range	566–759 nm	405–481 nm
Scan mode	stack, frame	
Scaling X	0.277 μm	
Scaling Y	0.277 μm	
Scaling Z	0.700 μm	
Lasers	100% (optimal)	10% (optimal)
Gain	550 (optimal)	520 (optimal)
Objective	plan-apochromat 20×/0.8 M27	
Direction	single	
Z-stack slices interval	0.7 μm
  8. Define the Z-stack region by setting the first and the last plane to specify the scanning range. This step is crucial for obtaining a comprehensive three-dimensional image of the NMJ.
  9. Click the *Start Experiment* button to initiate the scanning process. The system will begin capturing the 3D image based on the defined Z-stack region and the set acquisition parameters.
  10. The figures are processed by orthogonal projection in the XY, XZ, and YZ planes (Figure 8A), and then exported into TIFF format using ZEN software.
    **Figure 8.**
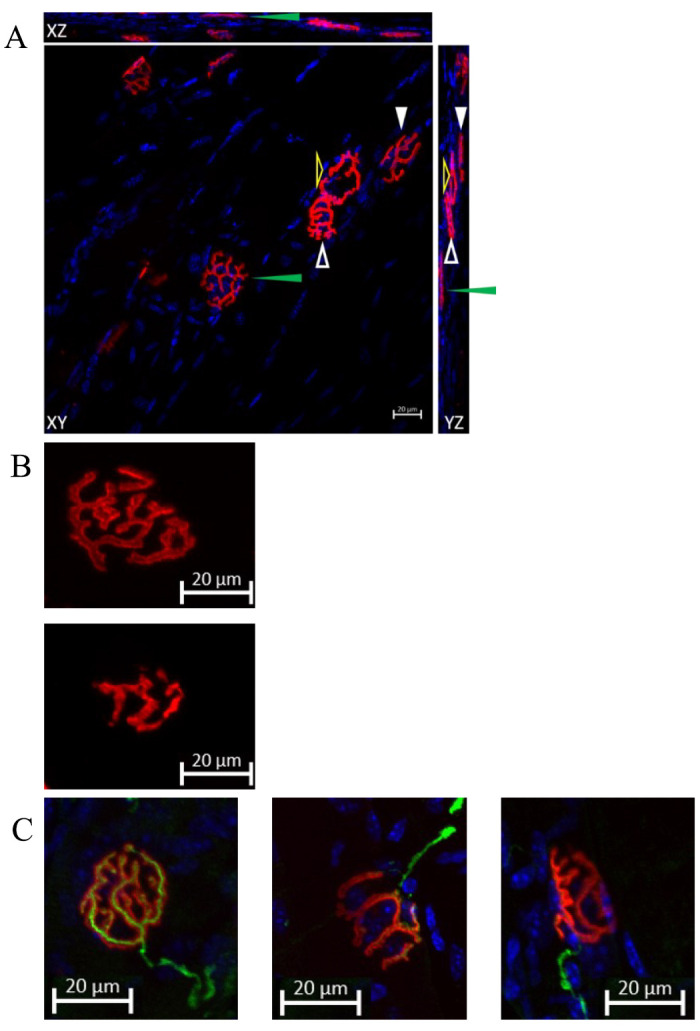
Representing data of co-staining of neuromuscular junctions (NMJs) and DAPI. (A) NMJs are labeled by Alexa-594-conjugated α-bungarotoxin, and the image is taken by confocal microscopy. The figure is processed with orthogonal projection in the XY, XZ, and YZ planes. Arrows of different forms point to specific NMJs on three planes. The green and white solid arrowheads indicate two distinct NMJs that are parallel to the XY plane. Although the NMJs pointed to by the white and yellow hollow arrowheads appear to be joined together in the XY plane, their independence can still be discerned by examining the YZ plane figure. Scale bar, 20 μm. A cleared representation of the NMJ in three-dimensional space is demonstrated in Video 1. (B) The comparison of normal NMJ and fragmented NMJ. The upper panel shows a normal morphology of an NMJ, characterized by a pretzel-like structure. The lower panel demonstrates a fragmented NMJ, which exhibits a decrease in area. This decrease will be discussed in the data analysis section. Both NMJs in the panels are parallel to the XY plane. Scale bar: 20 μm. (C) Representative images of co-staining of NMJ, neurofilament, and synaptophysin show a comparison of a fully innervated NMJ (left panel), a partially innervated NMJ (middle panel), and a denervated NMJ (right panel). A nerve terminal and motor endplate were deemed “fully innervated” if they overlapped by 50% or more. An overlap between 20% and 50% classified NMJs as “partially innervated.” Motor endplates without any coverage were identified as “denervated” NMJs [8]. Scale bar, 20 μm.
    **Video 1.**
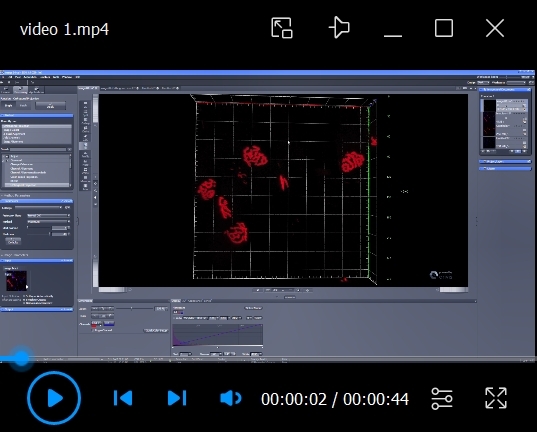
Representation of the three-dimensional (3D) structure of the neuromuscular junction in 3D space

## Data analysis

- **Image type conversion**
  1. Launch the ImageJ software.
  2. Go to *File* > *Open* and select the image file you want to work with.
  3. Press *type* in the list and select *8 bit* to convert the image.
- **Scale setting**
  1. Select the *Straight-Line tool* from the toolbar (it looks like a diagonal line).
  2. Draw a line on the image along a known distance (for example, if you have a scale bar on your image, draw the line along the length of the scale bar).
  3. Go to *Analyze* > *Set scale*.
  4. In the *Set Scale* dialog box, the *Distance* in pixels will be automatically filled in based on the length of the line you drew.
  5. Enter the actual distance that the line represents in the *Known distance* field (e.g., 10 if the scale bar represents 10 μm).
  6. Select the appropriate unit of length from the *Unit of Length* field (e.g., micrometers, millimeters, etc.).
  7. If you want the scale to be applied to all future measurements in this image, check the *Global* option.
  8. Click *OK* to set the scale.
- **Threshold adjustment**
  1. Go to *Image* > *Adjust* > *Threshold* to open the Threshold dialog box.
  2. The Threshold dialog box displays a histogram of pixel intensities and allows you to set the lower and upper threshold values using sliders.
  3. Adjust the sliders to include the range of pixel intensities that you want to highlight. Pixels within this range will be highlighted in red, indicating the thresholded area. Ensure the connectivity of all pixels.
  4. Press the *Apply* bottom; this will convert the highlighted regions into a binary image.
- **NMJ area measurement**
  1. Click on the *Wand tool* in the ImageJ toolbar (it looks like a magic wand) (Figure 9).
    **Figure 9.**
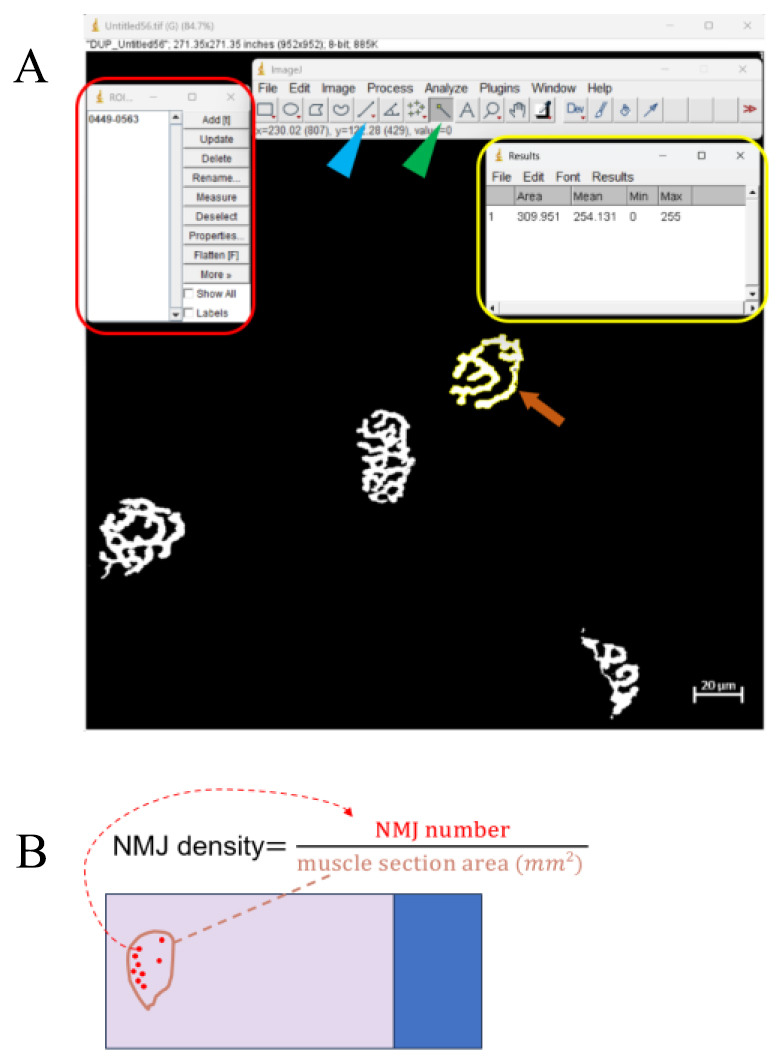
Representative example of neuromuscular junction (NMJ) quantification. (A) The red frame indicates the ROI manager dialog box that appears after pressing Ctrl + T. The yellow frame indicates the result window displaying area data after clicking *Measure* in the ROI manager dialog box. The orange arrow points to the selected NMJ after threshold adjustment. The blue and green arrowheads point to the *straight-line tool* and the *wand tool*, respectively. (B) Schematic figure that illustrates the formula for calculating NMJ density. Calculating the NMJ density is another method for analyzing NMJ physiology. The NMJ number is counted under the 10× objective lens of the microscope. The NMJ density is calculated by dividing the NMJ number by the muscle section area in mm^2^.
  2. Click inside the region you want to analyze. The Wand tool will automatically select the contiguous area with similar pixel values.
  3. Press Ctrl + T to add the selection to the ROI Manager. This will open the ROI Manager dialog box showing all selected regions.
  4. In the ROI Manager dialog box, select the region of interest from the list (Figure 9).
  5. Click *Measure* in the ROI Manager dialog box. This will open the results window, displaying the measurements of the selected region, including the area. (If there is a hollow area in the NMJ, its area needs to be deducted.)
- **NMJ area statistic**
  1. Calculate the mean value of the NMJ area in mice. Having at least 30 NMJs per mouse and the inclusion of at least three mice is critical: according to the central limit theorem, the distribution of sample means will be approximately normally distributed if the sample size is large enough (typically n ≥ 30). This normality is crucial for the validity of the T-test, which assumes a normally distributed data set. Moreover, including at least three mice ensures that the findings are not specific to a single mouse but are generalizable across multiple individuals.
  2. Set up a column table of GraphPad Prism 8.0.2 software and paste the data for the area of the NMJs.
  3. Go to *Analyze* in the menu bar and choose *t-test (and nonparametric tests)* in the submenu.
  4. Select unpaired t-test. If needed, select options for assuming equal variances or not (e.g., Welch's correction).
  5. Click *OK*. GraphPad Prism will provide the t-value, degrees of freedom, p-value, and confidence intervals.

## Validation of protocol

Two published papers can validate the measurements of NMJs:

1. Our previously published paper, “Muscle-restricted nuclear receptor interaction protein knockout causes motor neuron degeneration through down-regulation of myogenin at the neuromuscular junction” [4], provides a representative image and quantification of NMJ area in gastrocnemius muscle in Figure 3.
2. The methods for quantifying the NMJ area and calculating the percentage of innervated endplates are demonstrated in Figure 6 of Wong and Martin's 2010 publication, “Skeletal muscle-restricted expression of human SOD1 causes motor neuron degeneration in transgenic mice” [9].

## General notes and troubleshooting

**General notes**

1. The limitation of this protocol is that it still requires the dissection of mice, so it is impossible to monitor the physiological status of the NMJ in living mice in real-time.
2. Soleus and tibialis anterior muscles are acceptable for this protocol; however, they are relatively small, making it challenging during cryosectioning and difficult to visualize a sufficient number of NMJs, especially in the soleus. The best way to visualize NMJs in these muscles is to perform a whole-mount immunofluorescence experiment. This method does not require cryosectioning but involves extensive steps.
3. The signal intensities of NMJs are an unfavorable parameter for researchers to analyze, as signal intensity varies due to the three-dimensional conformation.

**Troubleshooting**

**Table:** 

	Cause	Solution
Tissue damage during dissection	Excessive force or improper handling.	Practice gentle handling techniques and keep tissues moist with PBS during dissection.
Tissue cracking while cryosectioning	Inappropriate freezing or blade dullness.	a. Use less liquid nitrogen to avoid flooding the mold. b. Use a new blade for cryosectioning.
Weak or no staining signal	Insufficient concentration of α-BTX applied.	a. Increase the concentration of α-BTX applied. b. When staining with antibodies for neurofilament or synaptophysin, permeabilize tissues with 0.1% Triton X-100 in PBS before the quenching procedure. c. Increase the detector gain during the scanning procedure of confocal microscopy.
